# Evaluation of a Heat Vulnerability Index on Abnormally Hot Days: An Environmental Public Health Tracking Study

**DOI:** 10.1289/ehp.1103766

**Published:** 2012-01-31

**Authors:** Colleen E. Reid, Jennifer K. Mann, Ruth Alfasso, Paul B. English, Galatea C. King, Rebecca A. Lincoln, Helene G. Margolis, Dan J. Rubado, Joseph E. Sabato, Nancy L. West, Brian Woods, Kathleen M. Navarro, John R. Balmes

**Affiliations:** 1 Environmental Health Sciences, School of Public Health, University of California–Berkeley, Berkeley, California, USA; 2 Massachusetts Department of Public Health, Bureau of Environmental Health, Boston, Massachusetts, USA; 3 Environmental Health Investigations Branch, California Department of Public Health, Richmond, California, USA; 4 Department of Health and Human Services, Maine Center for Disease Control and Prevention, Augusta, Maine, USA; 5 Department of Internal Medicine, School of Medicine, University of California–Davis, Davis, California, USA; 6 Office of Environmental Public Health, Oregon Health Authority, Portland, Oregon, USA; 7 Division of Environmental Health, Washington State Department of Health, Olympia, Washington, USA; 8 Environmental Health Epidemiology Bureau, New Mexico Department of Health, Santa Fe, New Mexico, USA; 9 Division of Occupational and Environmental Medicine, Department of Medicine, University of California, San Francisco, California, USA

**Keywords:** climate change, extreme heat, hospitalizations, mortality, vulnerable populations

## Abstract

Background: Extreme hot weather conditions have been associated with increased morbidity and mortality, but risks are not evenly distributed throughout the population. Previously, a heat vulnerability index (HVI) was created to geographically locate populations with increased vulnerability to heat in metropolitan areas throughout the United States.

Objectives: We sought to determine whether areas with higher heat vulnerability, as characterized by the HVI, experienced higher rates of morbidity and mortality on abnormally hot days.

Methods: We used Poisson regression to model the interaction of HVI and deviant days (days whose deviation of maximum temperature from the 30-year normal maximum temperature is at or above the 95th percentile) on hospitalization and mortality counts in five states participating in the Environmental Public Health Tracking Network for the years 2000 through 2007.

Results: The HVI was associated with higher hospitalization and mortality rates in all states on both normal days and deviant days. However, associations were significantly stronger (interaction *p*-value < 0.05) on deviant days for heat-related illness, acute renal failure, electrolyte imbalance, and nephritis in California, heat-related illness in Washington, all-cause mortality in New Mexico, and respiratory hospitalizations in Massachusetts.

Conclusion: Our results suggest that the HVI may be a marker of health vulnerability in general, although it may indicate greater vulnerability to heat in some cases.

There is considerable evidence of elevated mortality, and increasing evidence of increased morbidity, associated with heat waves and extreme hot weather conditions (Basu 2009; Basu and Samet 2002). Particular population subgroups are at increased risk of mortality during extreme heat, including the elderly (Fouillet et al. 2006; Medina-Ramon et al. 2006), people of lower socioeconomic status (Curriero et al. 2002; Naughton et al. 2002; Rey et al. 2009), people who live alone (Naughton et al. 2002; Semenza et al. 1996), people with less education (Medina-Ramon et al. 2006; O’Neill et al. 2003), people of races other than white (O’Neill et al. 2003; Schwartz 2005; Whitman et al. 1997), people with preexisting health conditions such as cardiovascular disease, diabetes, renal disease, nervous disorders, cerebrovascular disease, pulmonary conditions, and mental health conditions (Schwartz 2005; Semenza et al. 1996, 1999; Stafoggia et al. 2006, 2008), people without access to cooling devices such as air conditioning (Chestnut et al. 1998; Curriero et al. 2002; Semenza et al. 1996), and people in neighborhoods with less green space (Kilbourne et al. 1982; Tan et al. 2007).

Heat waves are projected to increase in frequency, severity, and duration in many parts of the world because of climate change (Meehl and Tebaldi 2004). Municipal interventions to prevent heat-related deaths have been shown to decrease mortality in subsequent heat events (Ebi et al. 2004; Fouillet et al. 2008; Naughton et al. 2002). There is some question, however, as to whether the most vulnerable populations are being reached by these interventions (Bassil and Cole 2010). Although there is an increased understanding by city governments of the need to have heat warning plans, they have also expressed their desire for more information to develop and implement such plans (Balbus et al. 2008; O’Neill et al. 2010).

Maps that identify which populations and areas within a city are most vulnerable to heat can help local governments allocate resources to the areas in greatest need (O’Neill et al. 2009). Reid et al. (2009) created a national heat vulnerability index (HVI) to locate populations vulnerable to heat at the submetropolitan level using variables associated with vulnerability in previous studies. Although others have created heat vulnerability maps for specific metropolitan areas (Johnson et al. 2009; Lindley et al. 2006; Loughnan et al. 2009; Rinner et al. 2009; Sister et al. 2009; Vescovi et al. 2005), the HVI is the only vulnerability map that is national in scope. The HVI suggests substantial variability in heat vulnerability across the United States as well as within metropolitan areas, but additional information is needed to confirm that higher rates of death and illness occur during abnormally hot days in areas that the HVI identifies as more vulnerable.

People adapt physiologically and technologically to the climate in which they live (Kinney et al. 2008). Heat waves earlier in the summer can be more hazardous to health than those later in the summer (Anderson and Bell 2011). Therefore, we investigated the impact of days that were much hotter than normal, for a given location and time of year, on hospitalization and mortality rates. Our study addresses the extent to which areas with higher HVI values experience higher morbidity and mortality on abnormally hot days.

This study is the result of a data linkage project within the Centers for Disease Control and Prevention’s (CDC) National Environmental Public Health Tracking (EPHT) Network in which researchers at the University of California–Berkeley (UCB) collaborated with public health professionals from EPHT programs in several states.

## Materials and Methods

Our study compared associations between the HVI and daily rates of morbidity and mortality on abnormally hot days and other days in five states that joined the Academic Center of Excellence research project being run by UCB as part of the EPHT Network. Although the HVI was calculated only in areas where air conditioning prevalence data were available (Reid et al. 2009), the analysis presented here includes 1,205 ZIP codes in California, 392 in Massachusetts, 20 in New Mexico, 119 in Oregon, and 212 in Washington that correspond to approximately 71%, 79%, 7%, 30%, and 40% of ZIP codes in each state, respectively [see Supplemental Material, Figure 1 (http://dx.doi.org/10.1289/ehp.1103766)].

**Figure 1:**
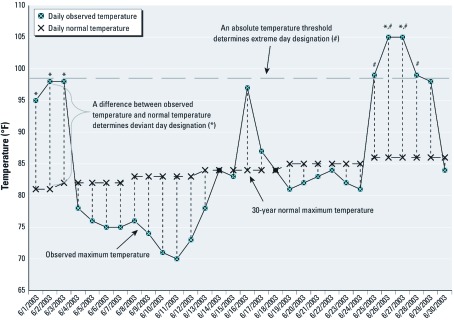
Deviant days and extreme days by daily observed temperature and daily normal temperature for one ZIP code for June 2003, as defined in “Materials and Methods.” For this specific ZIP code, a day was designated as a deviant day (*) if its observed maximum temperature was ≥ 14°F greater than the 30-year normal maximum temperature for that day, whereas a day was designated an extreme day (^#^) if its observed maximum temperature was ≥ 99°F.

For the years 2000 through 2007, each participating state assembled daily counts for hospitalization due to electrolyte imbalance [*International Classification of Diseases, Ninth Revision*, *Clinical Modification* (ICD-9-CM), code 276 (CDC 1979)], cardiovascular diseases (codes 390–398, 402, 404–429, 440–448), cerebrovascular disease (codes 430–438), respiratory illness (codes 460–519), nephritis and nephrotic syndrome (codes 580–589), acute renal failure (code 584), heat-related illness (code 992), and internal causes of hospitalization (all ICD-9-CM codes 0–799.9 except accidents, injuries, suicides, and homicides) and daily mortality counts, including all-cause mortality [all ICD-10 codes V01–Y98 (WHO 2007) except accidents, injuries, suicides, and homicides, excluding X30 (heat-related illness)], cardiovascular mortality (codes I00–I99), and respiratory mortality (codes J00–J99). These health outcomes have been analyzed in previous studies of extreme heat (Basu 2009; Knowlton et al. 2009; Ye et al. 2012). Daily counts for each outcome were calculated by searching all primary diagnoses and the first nine secondary diagnoses in hospital discharge and mortality data, similar to previous research (Knowlton et al. 2009). Data were obtained from the respective data stewards for each state. Mortality data come from the California Office of Vital Records, Center for Health Statistics (Sacramento, CA); Massachusetts Department of Public Health’s Registry of Vital Records and Statistics, Bureau of Vital Records and Health Statistics (Boston, MA); New Mexico Department of Health, Epidemiology and Response Division, State Center for Health Statistics (Santa Fe, NM); Oregon Health Authority, Public Health Division, Center for Health Statistic (Salem, OR); and Washington State Department of Health, Center for Health Statistics (Olympia, WA). Hospitalization data were obtained from the California Office of Statewide Health Planning and Development (Sacramento, CA); Massachusetts Division of Healthcare, Finance and Policy, Hospital Inpatient Discharge Database (Boston, MA); New Mexico Health Policy Commission, Office of Health Policy and Research, New Mexico Hospital Inpatient Discharge Data (Albuquerque, NM); Oregon Health Authority; and Washington State Department of Health (Olympia, WA), Center for Health Statistics, Comprehensive Hospitalization Abstract System (Salem, OR). These data were collected by the state partners and were not shared with the researchers at UCB.

The development of the HVI was previously described in Reid et al. (2009). Briefly, the HVI is composed of four factors derived from a principal components analysis (Table 1). Each factor has possible values of 1–6, with higher values denoting higher vulnerability. The HVI is the sum of the four factors (possible values of 4–24 for a given census tract). For this analysis, we calculated the HVI for each ZIP code as the area-weighted mean value of the census tracts that intersected with the ZIP code using the Geospatial Modeling Environment (Spatial Ecology LLC 2010); therefore, the HVI for each ZIP code is constant throughout our study. ZIP codes were assigned to weather stations based on both proximity and similarity of climate. First, we determined the 30-year (1971 through 2000) warm-season normal temperature for each ZIP code centroid in the study area based on 30-year warm-season normal data from the Oregon State University Parameter-Elevation Regressions on Independent Slopes Model (PRISM) Climate Group (2010) normals product. Next, we obtained data on 30-year daily normals for maximum temperature for all weather stations in the study states and contiguous states from the National Climatic Data Center (2010) CLIM84 daily normals product. We then assigned each study area ZIP code to a weather station, such that ZIP codes that contained a single weather station (7.7%) were assigned to that weather station and ZIP codes that contained more than one weather station (0.5%) were assigned to the station whose 30-year warm-season normal was the closest to the ZIP code’s PRISM-derived 30-year warm-season normal. For ZIP codes with no weather station (91.9%), we assigned them to the weather station (out of the three closest stations) with the 30-year warm-season normal that was closest to that of the ZIP code’s PRISM-derived 30-year warm-season normal. ZIP code assignment to monitors was done using ArcGIS (version 9.3; ESRI, Redlands, CA).

**Table 1:** Components of each factor in the HVI.

Factor	Main componentsa
1 Social/environmental	Percentage of population below the poverty line
	Percentage of population of a race other than white
	Percentage of population with less than a high school diploma
	Percentage of nongreen space
2 Social isolation	Percentage of population that live alone
	Percentage of population > 65 years of age that live alone
3 Air conditioning prevalence	Percentage of homes without central air conditioning
	Percentage of homes with no air conditioning of any kind
4 Preexisting health conditions	Percentage of population diagnosed with diabetes
	Percentage of population > 65 years of age
aThe factors were created using principal components analysis. Listed here are the variables that loaded most heavily on each factor. Reid et al. (2009) explained how the factors were created.	

For each day from 1 May through 30 September, for 2000 through 2007, we calculated the deviation of the daily maximum temperature from each weather station’s 30-year (1971 through 2000) daily normal maximum temperature. “Deviant days” were defined as days on which the deviation of maximum temperature from the 30-year normal was in the upper 5th percentile of the deviations for the 8 years of this study (2000 through 2007) for that weather station. “Extreme days” were defined as days in the upper 5th percentile of the absolute maximum temperature for the 8 years of this study (2000 through 2007) for a given weather station (Figure 1). Deviant and extreme days whose maximum temperature was < 85°F (29.4°C) were reclassified as nondeviant or nonextreme because we wanted to focus on the health effects of higher temperatures.

Each state partner calculated incidence rate ratios (RRs) for each hospitalization and mortality diagnosis using generalized estimating equation (GEE) Poisson regression clustering by ZIP code with an exchangeable working correlation matrix and the 2000 U.S. Census (population as an offset term) (U.S. Census Bureau 2002). GEE methods are robust to misspecification of the correlation matrix and to overdispersion. Daily counts of each health diagnosis category were regressed against HVI, deviant day, and the interaction between deviant day and the HVI while controlling for day of week, month, and daily maximum 8-hr ozone level. Similar models were run for extreme days. Separate models were also constructed to estimate the effects of HVI and deviant day or extreme day without adjustment for one another. Daily ground-level ozone measurements were obtained from the U.S. Environmental Protection Agency’s Air Quality System (2010) for all monitors and days in the study area and time period. We calculated the daily maximum 8-hr average ozone value in a 24-hr period starting at 0800 hours for each monitor. ZIP codes were assigned the daily ozone data from the nearest air quality monitor.

To investigate potential nonlinear relationships between HVI and hospitalizations and mortality, we modeled indicator variables for low, medium, and high HVI categories based on tertiles of the HVI distribution for all ZIP codes included in this analysis. All analyses were done using SAS (versions 9.1 and 9.2; SAS Institute Inc., Cary, NC) and R (version 2.11.1; R Project for Statistical Computing, Vienna, Austria). Statistical significance was determined as 95% confidence intervals (CIs) that did not cross 1, which assumes an α level of 0.05.

## Results

Mean HVI values were similar in each state (Table 2). Tertiles of HVI were driven heavily by the distribution of HVI values in California, which included 60% of the ZIP codes in our analysis. California and Washington each had a greater percentage of ZIP codes in the highest HVI tertile (38% and 40%, respectively) than the other states (5–20%). California had the highest daily mean counts of hospitalizations for each outcome. New Mexico had the fewest observations (ZIP code-days) of any state because it had only 20 ZIP codes for which an HVI value could be calculated. Hospitalizations for heat-related illness were rare, occurring on < 2% of ZIP code-days in each state. Means and standard deviations of daily mortality and morbidity counts were similar across all five states. Washington had the fewest days classified as deviant (Table 2) because it had more days that were categorized, initially, as deviant that had maximum temperatures < 85°F. Reclassification of deviant days to nondeviant ranged from 3.9% of days in New Mexico to 29.5% in Washington. No extreme days were reclassified in Massachusetts or New Mexico, but a high of 8.1% were reclassified in California. The days classified as deviant were most likely to be in May for California and New Mexico and in June for Massachusetts, Oregon, and Washington, whereas days classified as extreme were most likely to be in July in all states except Massachusetts, where most extreme days occurred in August (Table 3).

**Table 2:** Study variables for ZIP code-days by state.

Variable	CA (n = 1,474,920)	MA (n = 479,808)	NM (n = 24,480)	OR (n = 145,656)	WA (n = 259,488)
Mortality counts (mean ± SD)										
All cause		0.40 ± 0.71		0.29 ± 0.61		0.45 ± 0.78		0.29 ± 0.61		0.30 ± 0.60
Cardiovascular		0.16 ± 0.43		0.10 ± 0.34		0.15 ± 0.42		0.10 ± 0.33		0.11 ± 0.34
Respiratory		0.04 ± 0.20		0.03 ± 0.18		0.05 ± 0.22		0.03 ± 0.16		0.03 ± 0.17
Hospitalization counts (mean ± SD)										
Internal causes		6.85 ± 6.75		4.88 ± 5.42		5.97 ± 5.63		3.65 ± 4.15		4.01 ± 3.77
Cardiovascular diseases		1.90 ± 2.18		1.57 ± 2.01		1.23 ± 1.54		1.02 ± 1.43		1.09 ± 1.35
Respiratory diseases		1.42 ± 1.77		1.15 ± 1.62		1.14 ± 1.44		0.72 ± 1.12		0.86 ± 1.15
Cerebrovascular disease		0.35 ± 0.67		0.22 ± 0.52		0.23 ± 0.52		0.18 ± 0.46		0.18 ± 0.45
Electrolyte imbalance		0.90 ± 1.27		0.61 ± 0.61		0.68 ± 1.01		0.45 ± 0.82		0.56 ± 0.88
Heat-related illnesses		0.002 ± 0.05		0.001 ± 0.03		0.001 ± 0.03		0.001 ± 0.03		0.001 ± 0.02
Nephritis and nephritic syndrome		0.38 ± 0.76		0.28 ± 0.28		0.06 ± 0.40		0.19 ± 0.50		0.22 ± 0.53
Acute renal failure		0.20 ± 0.49		0.16 ± 0.44		0.13 ± 0.38		0.10 ± 0.34		0.12 ± 0.37
Exposure variables (%)a										
Deviant days		5.11		4.59		5.39		4.79		3.97
Extreme days		5.39		5.88		6.20		5.47		5.50
Vulnerability variables (mean ± SD)										
HVI (continuous)		14.5 ± 1.74		13.3 ± 1.38		13.3 ± 1.20		13.9 ± 1.13		14.5 ± 1.58
Social/environmental (factor 1)		3.49 ± 0.83		2.74 ± 0.68		3.25 ± 0.64		2.80 ± 0.48		2.85 ± 0.51
Social isolation (factor 2)		2.99 ± 0.79		3.53 ± 0.69		3.68 ± 0.92		3.37 ± 0.60		3.39 ± 0.85
Air conditioning prevalence (factor 3)		4.21 ± 1.14		4.01 ± 0.03		4.00 ± 0.00		5.00 ± 0.003		5.57 ± 0.77
Preexisting conditions (factor 4)		3.83 ± 0.58		2.98 ± 0.69		2.40 ± 0.48		2.73 ± 0.59		2.67 ± 0.66
HVI (categorical)		2.12 ± 0.81		1.57 ± 0.67		1.55 ± 0.59		1.82 ± 0.74		2.16 ± 0.78
Low (%)		27.9		53.1		50.0		38.5		24.1
Middle (%)		34.1		36.5		45.0		41.8		36.3
High (%)		37.9		10.4		5.0		19.7		39.6
8-hr ozone (ppm)		0.05 ± 0.02		0.04 ± 0.02		0.06 ± 0.01		0.03 ± 0.01		0.03 ± 0.01
Abbreviations: CA, California; MA, Massachusetts; NM, New Mexico; OR, Oregon; WA, Washington. aThe percentages for deviant days and extreme days by state can be > 5% because the temperature was equal to the 95th percentile value on many ZIP code-days.										

**Table 3:** Percentage of deviant days and extreme days in each month by state from May through September 2001 through 2007 (n = number of deviant days or extreme days).

State	May	June	July	August	September
Deviant days										
CA (n = 70,224)		38.5a		16.6		14.5		6.1		24.3
MA (n = 21,659)		26.9		31.0a		5.6		18.1		18.4
NM (n = 1,294)		44.1a		6.5		11.1		24.0		14.3
OR (n = 6,603)		20.3		33.5a		22.5		11.2		12.5
WA (n = 10,028)		13.3		33.3a		31.0		10.1		12.3
Extreme days										
CA (n = 74,388)		5.8		11.9		33.5a		23.1		25.7
MA (n = 27,661)		6.4		20.8		29.0		38.9a		4.9
NM (n = 1,479)		4.3		19.6		60.9a		14.8		0.3
OR (n = 7,909)		4.2		15.6		39.6a		31.7		10.0
WA (n = 15,063)		4.4		19.2		43.0a		26.8		6.7
Abbreviations: CA, California; MA, Massachusetts; NM, New Mexico; OR, Oregon; WA, Washington. aState’s month, with the highest percentage of deviant or extreme days.										

**Figure 2:**
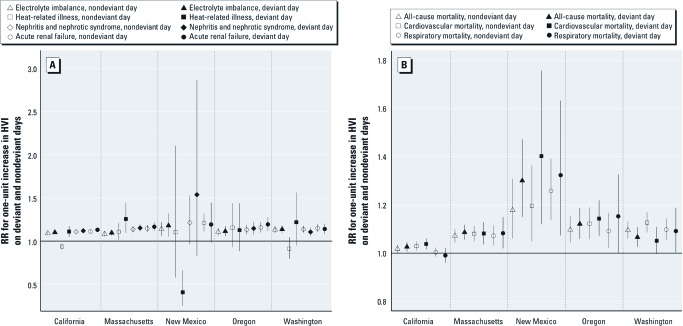
RRs for a one-unit increase in the HVI for five hospitalization diagnoses (*A*) and three mortality categories (*B*), by state, on nondeviant and deviant days.

On nondeviant days, the HVI was associated with higher rates of hospitalizations for electrolyte imbalance, acute renal failure, respiratory hospitalizations, and all-cause and cardiovascular mortality (*p* < 0.05) in all five states, and for nephritis and nephrotic syndrome in all states but New Mexico [Figure 2; see also Supplemental Material, Table 1 (http://dx.doi.org/10.1289/ehp.1103766)]. The magnitude of the estimated effect of HVI on health was essentially the same on deviant and nondeviant days for almost all outcomes in all states, an indication that living in a ZIP code with a higher HVI score denotes increased risk of mortality or hospitalization regardless of how hot the day is. However, some evidence of effect modification by deviant day was observed for heat-related illness, specifically in California [RRs (95% CIs): nondeviant day, 0.94 (0.90, 0.97); deviant day, 1.11 (1.05, 1.17)], Washington [nondeviant day, 0.91 (0.80, 1.04); deviant day, 1.22 (0.95, 1.56)], and Massachusetts [nondeviant day, 1.11 (1.01, 1.21); deviant day, 1.26 (1.10, 1.44); Figure 2A; see also Supplemental Material, Table 1 (http://dx.doi.org/10.1289/ehp.1103766)]. In Oregon, rates for this outcome show no difference between nondeviant and deviant days, whereas in New Mexico, there was a significant decrease in heat-related hospitalizations on deviant days for a one-unit increase in HVI. A significant interaction was also found for all-cause mortality in New Mexico [RRs (95% CIs): nondeviant day, 1.18 (1.06, 1.31); deviant day, 1.30 (1.15, 1.47); Figure 2B]. We also found statistically significant interaction terms for respiratory hospitalizations in Massachusetts and for electrolyte imbalance, nephritis and nephrotic syndrome, and acute renal failure in California; however, the magnitudes of the differences in estimated effects for deviant days and nondeviant days were small [Figure 2A; see also Supplemental Material, Table 1 (http://dx.doi.org/10.1289/ehp.1103766)]. The association between HVI and cardiovascular mortality was stronger on nondeviant days than on deviant days in Washington [RRs (95% CIs): nondeviant day, 1.13 (1.09, 1.17); deviant day, 1.05 (1.00, 1.11); Figure 2B]. The patterns for Oregon, except for heat-related illness, were similar to those in the other states where we had more power to see an effect.

Analysis of extreme days showed almost the same effect estimates and similar patterns to that observed with deviant days [see Supplemental Material, Table 2 (http://dx.doi.org/10.1289/ehp.1103766)]. The main difference is that for extreme days, there was consistency across all states for heat-related illness, with higher risk on extreme days compared with nonextreme days for a one-unit increase in the HVI.

Deviant days were identified as extreme days about half the time in most states, with the lowest percentage of agreement in New Mexico (21.7% of deviant days were extreme days) and the highest in Washington (81.4% of deviant days were extreme days). Given the relatively low agreement, it is interesting that, for the most part, there were no large differences in effect estimates for deviant days compared with extreme days in models without the HVI [see Supplemental Material, Table 3 (http://dx.doi.org/10.1289/ehp.1103766)].

When we analyzed tertiles of the HVI, we did not find evidence against the use of HVI as a continuous variable in our analyses (data not shown); however, because our study examined only five states, this should be further investigated in future studies.

## Discussion

The main purpose of our study was to investigate whether people living in more heat-vulnerable ZIP codes, as characterized by the HVI, experienced higher rates of mortality and morbidity during abnormally hot days than during other days. Overall, our findings indicate that HVI was consistently associated with most health outcomes on both normal (nondeviant days) and abnormally hot (deviant) days. Therefore, HVI may be a good indicator of overall health vulnerability, independent of exposure to heat. Given that the HVI incorporates many variables that are known to be strong determinants of ill health, this is not surprising. However, in at least some of the states, health outcomes were more strongly associated with HVI on abnormally hot days than on other days, especially for heat-related illness.

We found stronger associations between HVI and cardiovascular mortality on nonextreme days than on extreme days and between HVI and all-cause mortality on nondeviant days than on deviant days in Washington. This could be due to residual seasonal confounding because cardiovascular mortality is lowest in the middle of the summer, when deviant and extreme days were most likely to occur for Washington. The finding of a similar effect for heat-related hospitalizations in New Mexico could be due to small numbers.

No significant interactions were found in Oregon, which could imply that the HVI is not predictive of increased risk of adverse health effects on abnormally hot days in all parts of the country. Oregon had the second fewest ZIP codes, and, for some health outcomes, daily counts were low, so null findings might have been due to lower statistical power. However, the general pattern of higher RRs on deviant compared to nondeviant days for most health outcomes is consistent with our other findings.

Most previous studies of heat-related health effects have used time-series methods to determine whether day-to-day changes in temperature are associated with changes in health effects. To have sufficient power to test their hypotheses, these studies have aggregated health outcome data over larger spatial areas (e.g., county or populated metropolitan areas). The purpose of the HVI, however, is to identify the most vulnerable neighborhoods within a metropolitan area. Therefore, spatial aggregation beyond the ZIP code level was not appropriate for our analysis. Our recalculation of the HVI from census tracts to ZIP codes could have slightly changed the relationships observed, had health data been available at the census tract level.

This study is, to our knowledge, the first to investigate deviation of maximum temperature from the daily 30-year normal maximum temperature as a heat exposure metric. One of the primary benefits of this heat metric is that it captures the health effects of abnormally hot days earlier in the warm season that would have been missed by investigating the effect of the hottest days using absolute temperature. Temperature thresholds above which adverse health outcomes increase have been used in previous studies; some of these thresholds were determined statistically, whereas other studies used an *a priori* cut-point of the 95th or 99th percentile of temperature (Basu 2009). Our analysis used the 95th percentile of deviant maximum temperature for 8 years of warm seasons, 2000 through 2007, by weather station to attain an estimate of a local threshold temperature for each location. We found some differences between estimated effects of HVI on deviant days versus extreme days, but results were consistent overall. The reason for introducing the concept of a “deviant” day was to allay a concern that our analysis could have residual seasonal confounding if we used only extreme days because they are most likely to fall in July and August (Table 3), when outcomes such as cardiovascular hospitalizations are lowest and extreme days are most likely. Deviant days are less likely to follow the temporal pattern; therefore, we were less concerned about temporal confounding than with extreme days. Time-series analysis of these exposure metrics could shed more light on whether deviance from normal temperature is a valid or more precise way to estimate the effect of high temperatures on health.

Previous efforts to create heat vulnerability maps (e.g., Johnson et al. 2009; Loughnan et al. 2009) have differed in the variables selected, the methods used to combine the variables, and whether they have been evaluated for their ability to predict spatial variability in health risks. Further analysis is needed to determine whether cities differ with regard to important predictors of heat-related morbidity or mortality, or if variation in results among studies is due to methodological differences. Over time, patterns of heat vulnerability are likely to change, for example, because of shifts in aging population distributions or changing patterns of underlying disease, necessitating periodic reevaluation of vulnerability maps.

The HVI was created using a principal components analysis for all areas in the United States for which data on component variables were available. If the HVI were created instead based on data from a single metropolitan location or state, the resulting HVI values might change because of different variable loadings on the factors. Indeed, a study conducted in Phoenix, Arizona, found that although the factors had different combinations of the same 10 vulnerability variables used in the national HVI, this locally derived HVI did predict locations with heat-related deaths (Harlan 2010). Although evidence exists of local differences in vulnerability to heat (Basu 2009), future research should investigate whether specific subcomponents of the HVI are more important in some regions of the country than in others. It is likely unreasonable to expect every local health department to create its own heat vulnerability map, and therefore, a national HVI created through freely available national data sets is useful.

Our study was limited by the number of states that participated in this project. The states involved in this study do not represent the diversity of climates or vulnerability patterns that exist throughout the United States. Additionally, because of confidentiality concerns on the part of data providers, health data were not able to be shared with UCB, and thus we could not combine the data into one “national” analysis. Our research was also limited by the few ZIP codes for which we could analyze data in each state because of where the original HVI had been calculated. Further research that includes more states and allows for sharing of data could further the understanding of spatial vulnerability to heat and the usefulness of a national HVI for climate change adaptation purposes.

## Conclusions

Climate change will likely exacerbate health risks that populations already experience, including, but not limited to, health effects related to exposure to extreme heat. Mapping the locations of populations that are vulnerable to climate-change–related risks facilitates planning of interventions to prevent adverse health events. Our results suggest that the HVI can be used to identify areas with increased risks of adverse health outcomes in general, and that it may identify areas at increased risk of heat-related illness and possibly other heat-related outcomes on abnormally hot days. Heat may exacerbate preexisting health disparities associated with the HVI. Deviant days and extreme days were found to capture health effects associated with heat similarly well. Further investigation is warranted to assess whether the HVI indicates increased risk of health effects in association with different heat exposure metrics and whether the HVI should be modified for different regions of the country. Targeting resources toward decreasing inequities in vulnerability now may increase communities’ resilience to multiple hazards to health in the future.

## Supplemental Material

(618 KB) PDF
