# Supplementary material for: Evaluation of a Heat Vulnerability Index on Abnormally Hot Days: An Environmental Public Health Tracking Study
Source: Environ Health Perspect. 2012 Jan 31;120(5):715–20. doi: 10.1289/ehp.1103766 (PMC3346770; doi:10.1289/ehp.1103766)
Supplement: (618 KB) PDF [file ehp.1103766.s001.pdf]

Supplemental Material

Title: "Evaluation of a Heat Vulnerability Index on Abnormally Hot Days: an Environmental Public Health Tracking Study"

Authors:
Colleen E. Reid, Jennifer K. Mann, Ruth Alfasso, Paul B. English, Galatea C. King, Rebecca A. Lincoln, Helene G. Margolis, Dan J. Rubado, Joseph E. Sabato, Nancy L. West, Brian Woods, Kathleen M. Navarro, John R. Balmes

Table of Contents:

Supplemental Material, Figure 1: Map of Study Zip Codes ……………………………………...2

Supplemental Material, Table 1: Rate Ratios for a one-unit increase in HVI on non-deviant and deviant days………………………………………..3

Supplemental Material, Table 2: Rate Ratios for a one-unit increase in HVI on non-extreme and extreme days………………………………………4

Supplemental Material, Table 3: Comparison of Rate Ratios for on Deviant and Extreme Day in Separate Models that Do Not Include the HVI………5

Supplemental Material, Figure 1: Map of Study Zip Codes

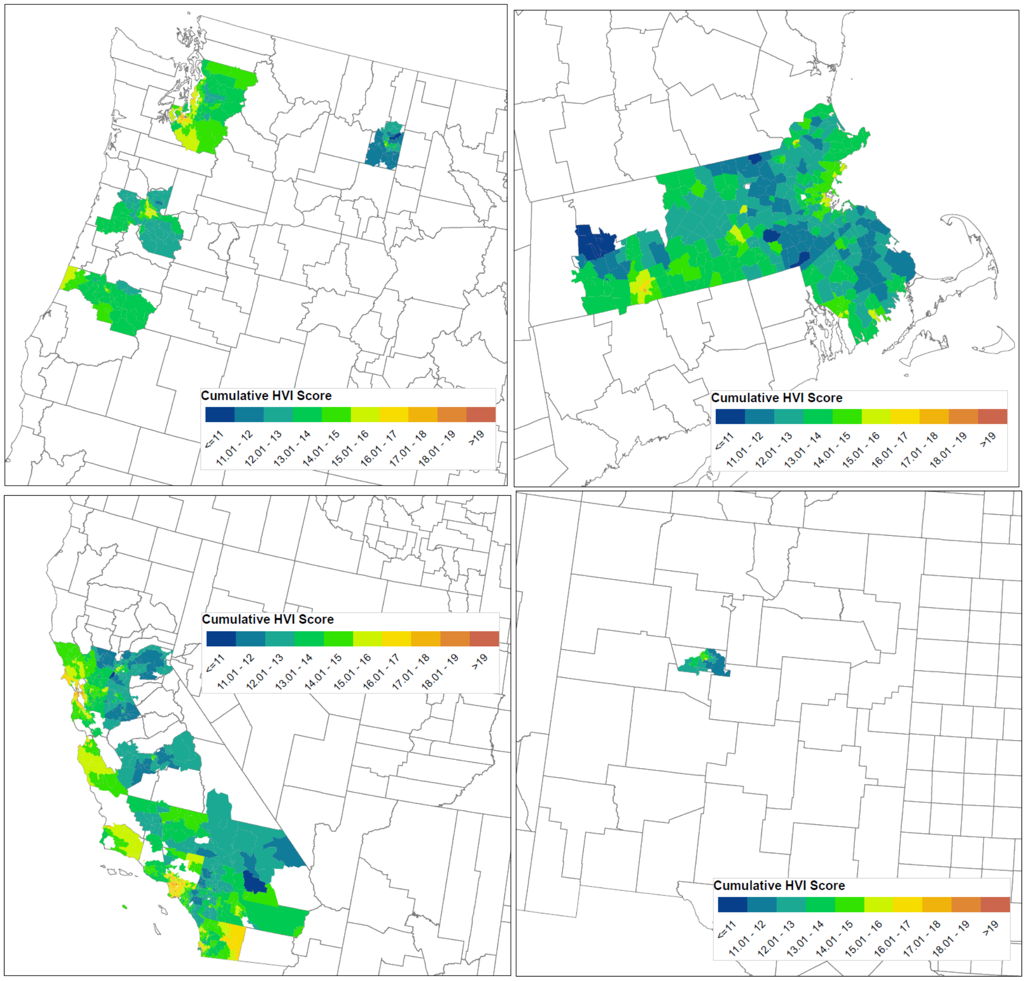

Supplemental Material, Table 1: Rate Ratios for a one-unit increase in HVI on non-deviant and deviant days

	California		Massachusetts		New Mexico		Oregon		Washington	
	Non-Deviant day	Deviant day	Non-Deviant day	Deviant day	Non-Deviant day	Deviant day	Non-Deviant day	Deviant day	Non-Deviant day	Deviant day
Hospitalization										
cerebrovascular	1.09(1.07,1.11)	1.10(1.08,1.12)	1.07(1.04,1.10)	1.07(1.03,1.10)	1.11(1.01,1.22)	1.20(0.96,1.50)	1.05(0.99,1.11)	1.09(1.00,1.18)	1.11(1.08,1.15)	1.12(1.08,1.17)
cardiovascular	1.04(1.02,1.06)	1.04(1.03,1.06)	1.00(0.98,1.02)	1.01(0.98,1.03)	1.07(0.95,1.20)	1.08(0.91,1.28)	1.03(0.97,1.09)	1.01(0.96,1.08)	1.06(1.02,1.09)	1.07(1.03,1.11)
electrolyte imbalance	1.09(1.07,1.11)	1.10(1.08,1.12)	1.08(1.05,1.11)	1.10(1.07,1.13)	1.14(1.06,1.22)	1.18(1.05,1.32)	1.11(1.06,1.15)	1.11(1.06,1.17)	1.13(1.09,1.17)	1.14(1.09,1.18)
heat-related illness	0.94(0.90,0.97)	1.11(1.05,1.17)	1.11(1.01,1.21)	1.26(1.10,1.44)	1.10(0.58,2.10)	0.41(0.25,0.66)	1.15(0.93,1.44)	1.12(0.88,1.44)	0.91(0.80,1.04)	1.22(0.95,1.56)
internal causes	0.99(0.98,1.00)	0.99(0.98,1.01)	0.97(0.96,0.99)	0.98(0.96,1.00)	0.99(0.93,1.05)	0.99(0.92,1.06)	0.97(0.93,1.01)	0.96(0.92,1.00)	0.99(0.95,1.02)	0.99(0.95,1.03)
nephritis and nephrotic syndrome	1.11(1.09,1.13)	1.12(1.10,1.14)	1.14(1.10,1.17)	1.15(1.11,1.19)	1.21(0.96,1.53)	1.54(0.82,2.87)	1.13(1.08,1.19)	1.15(1.08,1.22)	1.14(1.10,1.18)	1.11(1.06,1.16)
renal failure	1.11(1.09,1.13)	1.13(1.11,1.15)	1.15(1.11,1.19)	1.17(1.12,1.22)	1.21(1.11,1.32)	1.19(0.98,1.45)	1.16(1.10,1.22)	1.19(1.12,1.27)	1.15(1.11,1.19)	1.14(1.08,1.20)
respiratory	1.04(1.02,1.06)	1.04(1.02,1.06)	1.10(1.07,1.12)	1.11(1.08,1.14)	1.08(1.00,1.16)	1.11(1.01,1.21)	1.07(1.01,1.13)	1.07(1.01,1.15)	1.08(1.04,1.11)	1.09(1.05,1.12)
Mortality										
all-cause	1.02(1.00,1.03)	1.03(1.01,1.05)	1.07(1.04,1.10)	1.09(1.06,1.12)	1.18(1.06,1.31)	1.30(1.15,1.47)	1.10(1.04,1.15)	1.12(1.06,1.19)	1.09(1.06,1.13)	1.07(1.03,1.11)
cardiovascular	1.03(1.01,1.05)	1.04(1.02,1.06)	1.08(1.05,1.11)	1.08(1.04,1.13)	1.20(1.05,1.36)	1.40(1.12,1.76)	1.12(1.06,1.19)	1.14(1.07,1.22)	1.13(1.09,1.17)	1.05(1.00,1.11)
respiratory	1.00(0.99,1.02)	0.99(0.96,1.02)	1.07(1.03,1.11)	1.08(1.02,1.15)	1.26(1.14,1.39)	1.32(1.07,1.63)	1.09(1.02,1.17)	1.15(1.00,1.33)	1.10(1.05,1.14)	1.09(1.00,1.19)

Models included the following variables: HVI, deviant day, the interaction between deviant day and the HVI 8-hourozone, month and day of week.

Supplemental Material, Table 2: Rate Ratios for a one-unit increase in HVI on non-extreme and extreme days

	California		Massachusetts		New Mexico		Oregon		Washington	
	Non-extreme Day	Extreme Day	Non-extreme Day	Extreme Day	Non-extreme Day	Extreme Day	Non-extreme Day	Extreme Day	Non-extreme Day	Extreme Day
Hospitalizations										
cerebrovascular	1.09(1.07,1.11)	1.09(1.08,1.11)	1.07(1.04,1.09)	1.08(1.05,1.12)	1.12(1.01,1.23)	1.09(0.90,1.33)	1.05(0.99,1.12)	1.06(0.98,1.15)	1.11(1.08,1.15)	1.11(1.07,1.16)
cardiovascular	1.04(1.02,1.06)	1.04(1.02,1.06)	1.00(0.98,1.02)	1.00(0.98,1.03)	1.08(0.96,1.21)	1.04(0.88,1.23)	1.03(0.97,1.09)	1.02(0.97,1.08)	1.06(1.02,1.09)	1.06(1.02,1.10)
electrolyte imbalance	1.09(1.07,1.11)	1.09(1.07,1.11)	1.08(1.05,1.11)	1.09(1.06,1.12)	1.14(1.06,1.22)	1.20(1.08,1.33)	1.11(1.06,1.15)	1.10(1.05,1.16)	1.13(1.09,1.17)	1.14(1.10,1.18)
heat-related illness	0.94(0.90,0.97)	1.13(1.07,1.19)	1.09(0.98,1.20)	1.21(1.07,1.37)	1.02(0.57,1.82)	1.17(0.28,4.82)	1.16(0.93,1.44)	1.19(0.90,1.56)	0.96(0.83,1.11)	1.03(0.84,1.27)
internal causes	0.99(0.98,1.00)	0.99(0.98,1.00)	0.97(0.96,0.99)	0.98(0.96,1.00)	0.99(0.93,1.05)	0.97(0.90,1.05)	0.97(0.93,1.01)	0.97(0.93,1.01)	0.99(0.95,1.03)	0.99(0.95,1.03)
nephritis and nephrotic syndrome	1.11(1.09,1.12)	1.11(1.09,1.13)	1.13(1.10,1.17)	1.17(1.13,1.21)	1.21(0.96,1.53)	1.37(0.78,2.40)	1.13(1.08,1.19)	1.13(1.06,1.19)	1.13(1.10,1.17)	1.13(1.08,1.19)
renal failure	1.11(1.09,1.13)	1.12(1.10,1.14)	1.14(1.11,1.18)	1.18(1.13,1.23)	1.20(1.11,1.31)	1.25(1.06,1.48)	1.16(1.10,1.22)	1.16(1.09,1.23)	1.15(1.10,1.19)	1.16(1.10,1.23)
respiratory	1.04(1.02,1.06)	1.04(1.02,1.06)	1.10(1.07,1.12)	1.11(1.08,1.13)	1.08(1.00,1.16)	1.06(1.00,1.13)	1.07(1.01,1.13)	1.06(1.00,1.13)	1.08(1.04,1.11)	1.09(1.05,1.13)
Mortality										
all-cause	1.02(1.00,1.03)	1.02(1.01,1.04)	1.07(1.04,1.10)	1.08(1.05,1.12)	1.19(1.07,1.32)	1.13(1.01,1.27)	1.10(1.04,1.15)	1.12(1.06,1.19)	1.09(1.06,1.13)	1.08(1.04,1.12)
cardiovascular	1.03(1.01,1.05)	1.04(1.01,1.06)	1.08(1.05,1.11)	1.08(1.04,1.12)	1.20(1.05,1.38)	1.09(0.89,1.33)	1.12(1.06,1.19)	1.17(1.10,1.24)	1.13(1.08,1.17)	1.08(1.03,1.13)
respiratory	1.00(0.98,1.02)	1.01(0.98,1.04)	1.07(1.03,1.11)	1.08(1.02,1.15)	1.27(1.16,1.40)	1.15(0.77,1.72)	1.09(1.02,1.16)	1.20(1.05,1.38)	1.10(1.06,1.14)	1.07(0.98,1.17)

Supplemental Material, Table 3: Comparison of Rate Ratios for on Deviant and Extreme Day in Separate Models that Do Not Include the HVI

	California Deviant Day	Extreme Day	Massachusetts Deviant Day	Extreme Day	New Mexico Deviant Day	Extreme Day	Oregon Deviant Day	Extreme Day	Washington Deviant Day	Extreme Day
Hospitalizations										
cerebrovascular	1.02(1.00,1.03)	1.01(0.99,1.02)	1.01(0.98,1.04)	1.00(0.97,1.03)	1.02(0.89,1.16)	0.94(0.80,1.10)	1.02(0.96,1.08)	0.99(0.94,1.05)	0.99(0.94,1.03)	0.98(0.94,1.02)
cardiovascular	1.00(0.99,1.01)	1.00(1.00,1.01)	1.01(1.00,1.02)	1.01(1.00,1.02)	1.02(0.95,1.09)	0.96(0.89,1.03)	1.04(1.01,1.07)	1.04(1.02,1.07)	1.02(0.99,1.04)	1.02(1.00,1.04)
electrolyte imbalance	1.05(1.04,1.06)	1.07(1.06,1.08)	1.06(1.04,1.08)	1.08(1.06,1.10)	1.01(0.95,1.07)	1.06(0.98,1.14)	1.04(1.00,1.07)	1.12(1.08,1.16)	1.06(1.03,1.10)	1.05(1.02,1.09)
heat-related illness	7.49(6.63,8.48)	7.08(6.25,8.01)	6.60(4.91,8.86)	6.31(4.93,8.06)	1.65(0.34,8.03)	1.07(0.38,3.03)	5.34(3.07,9.29)	3.69(1.89,7.18)	5.35(3.30,8.66)	5.54(3.58,8.58)
internal causes	1.00(1.00,1.00)	1.01(1.00,1.01)	1.01(1.00,1.01)	1.01(1.00,1.02)	1.03(1.00,1.06)	1.01(0.99,1.04)	1.02(1.01,1.04)	1.04(1.03,1.06)	1.01(1.00,1.02)	1.02(1.01,1.03)
nephritis & nephrotic syndrome	1.07(1.06,1.09)	1.13(1.11,1.15)	1.10(1.07,1.13)	1.05(1.02,1.08)	0.83(0.58,1.19)	0.96(0.60,1.56)	1.03(0.98,1.09)	0.99(0.94,1.04)	1.05(1.00,1.10)	1.02(0.98,1.06)
acute renal failure	1.07(1.05,1.09)	1.13(1.11,1.15)	1.07(1.04,1.11)	1.06(1.02,1.10)	1.01(0.88,1.16)	1.23(1.11,1.35)	1.02(0.95,1.10)	1.05(0.98,1.11)	1.07(1.01,1.14)	1.06(1.01,1.11)
respiratory	1.01(1.00,1.01)	1.01(1.00,1.02)	1.02(1.00,1.03)	1.01(1.00,1.03)	1.05(0.98,1.11)	0.99(0.95,1.03)	1.02(0.99,1.05)	1.03(1.00,1.07)	1.02(1.00,1.05)	1.03(1.01,1.06)
Mortality										
all-cause	1.05(1.04,1.07)	1.06(1.04,1.07)	1.05(1.02,1.07)	1.03(1.00,1.05)	1.08(0.99,1.18)	0.97(0.90,1.06)	1.00(0.96,1.05)	0.98(0.93,1.03)	1.03(0.99,1.07)	1.02(0.98,1.05)
cardiovascular	1.06(1.04,1.09)	1.06(1.03,1.08)	1.04(0.99,1.09)	1.03(0.99,1.07)	1.00(0.85,1.16)	1.04(0.89,1.23)	0.98(0.89,1.08)	0.94(0.86,1.02)	1.07(1.01,1.15)	1.00(0.95,1.06)
respiratory	1.05(1.01,1.09)	1.05(1.01,1.09)	1.10(1.01,1.19)	1.03(0.96,1.10)	1.09(0.87,1.36)	0.90(0.62,1.31)	1.02(0.87,1.20)	1.04(0.88,1.22)	0.97(0.86,1.11)	0.99(0.89,1.10)
